# SIRT1 Activators: The Evidence STACks up

**DOI:** 10.18632/aging.100534

**Published:** 2013-03-08

**Authors:** Sita Kugel, Raul Mostoslavsky

**Affiliations:** The Massachusetts General Hospital Cancer Center, Harvard Medical School, Boston MA 02114 USA

SIRT1 is the mammalian ortholog of silent information regulator 2 (Sir2) found in *Saccharomyces cerevisiae* and functions as a NAD^+^-dependent deacetylase. SIRT1 appears to promote healthy aging and is implicated in the prevention of many age-related pathologies [1]. At the cellular level, SIRT1 controls lipid and glucose homeostasis, DNA repair and apoptosis, circadian clocks, inflammation and mitochondrial biogenesis. The biological effects of SIRT1 are mediated by its ability to deacetylate several key transcription factors such as Peroxisome proliferator-activated receptor-ϒ coactivator 1 alpha (PGC-1α), p53, and FOXO proteins [2].

For many years there has been interest in characterizing sirtuin-activating compounds (STACs) that can modulate the ability of SIRT1 to deacetylate substrate proteins. These compounds would have the potential of reducing the incidence of multiple age-related diseases. Resveratrol and a series of chemically unrelated synthetic molecules have been described as potential STACs [3-7]. The original reports demonstrated activation by using an enzyme assay that contained a fluorescently labeled peptide substrate. However, the validity of these findings was challenged when others demonstrated that activation was dependent on the presence of the fluorophore on the substrate. Multiple studies followed, some in favor and some against [8-10]. However, two new studies, one by Hubbard et al., (2013) in this month's issue of Science and a second one by Lakshminarasimhan et al., (2013) in recent of Aging, appear to elegantly resolve this controversy.

**Figure 1:**
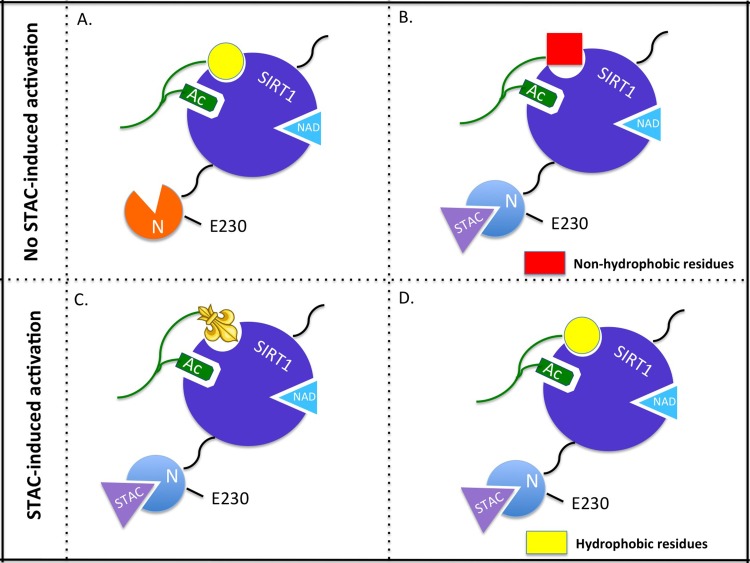
Model of allosteric activation of SIRT1 by sirtuin activating compounds (STACs). (**A**) SIRT1 acting on a substrate with a hydrophobic signature (yellow) in the absence of a STAC. (**B**) Binding of a STAC alters the N-terminal structure of SIRT1 but the absence of hydrophobic residues C-terminal to the acetyl-lysine on the substrate prevents activation by STACs. (**C**) The aminomethycoumarin group on the Fluor-de-Lys peptide substrate mimics hydrophobic residues of natural substrates, facilitating activation by STACs. (**D**) Substrate contains hydrophobic residues C-terminal to the acetyl-lysine thus allowing STAC-induced activation. Mutation of E230 allows STACs to bind to SIRT1 but abolishes STAC-mediated allosteric activation.

Lakshminarasimhan and colleagues used a mammalian acetylome microarray system to determine whether natural deacetylation sites can respond to resveratrol-dependent SIRT1 activation. After testing almost 7,000 peptides, surprisingly, very few of them exhibited increased deacetylation in the presence of resveratrol. They found that deaceylation by SIRT1 was preferentially activated when the substrates contained large, mainly hydrophobic residues at several positions C-terminal to the acetyl-lysine. These results provided a clear potential explanation of why the Fluor-de-Lys fluorophore, which is bulky and hydrophobic, may replace the peptide chain immediately C-terminal to the acetyl-lysine, likely mimicking a natural hydrophobic residue.

Indeed, Hubbard and colleagues provided strong support for such a model, and extended the above findings by demonstrating that a series of STACs, including the Sirtris compounds and resveratrol, directly activate SIRT1 through an allosteric mechanism. They first determined that the fluorophore caused activation only when it was directly adjacent (+1) to the acetyl-lysine. Similar to the findings of Lakshminarasimhan et al., (2013) the fluorophore moieties were dispensable if replaced with naturally occurring hydrophobic amino acids. For instance, native peptide sequences of PGC-1α and FOXO3a supported activation by STACs which was dose-dependent and mediated through a lowering of peptide K_m_. When the aromatic or hydrophobic amino acids at position +1 or +6 of PGC-1α or +1 of FOXO3a were mutated to alanine, activation by STACs was blocked. To establish the mechanism of activation, they screened SIRT1 mutants that were unable to be activated by resveratrol. Substitution of a glutamate for lysine at position 230 in the structured N-terminal domain attenuated (or abolished) SIRT1 activation by 117 chemically diverse STACs independent of the substrate. Altering this residue did not reduce the basal catalytic activity of SIRT1 or significantly change the V_max_ or K_m_ of several substrates but rather specifically inhibited activation by STACs. Finally, they reconstituted SIRT1 KO myoblasts with wild-type or mutant SIRT1 and observed STAC-induced increases in mitochondrial mass and ATP content only in wild-type-reconstituted myoblasts, thus demonstrating that the effect of STACs on mitochondrial function is clearly SIRT1-dependent and direct. This work elegantly describes a SIRT1-dependent mechanism of “assisted allosteric activation” for STACs, providing a putative molecular explanation for the previous controversy.

The findings by both groups that only a small subset of SIRT1 substrates have increased deacetylation by SIRT1 in the presence of STACs is promising for future therapeutic intervention strategies, since the selectivity of STACs could be far more targeted than previously anticipated. For instance, the SIRT1 substrates PGC-1α and FOXO3a, but not p53, have hydrophobic residues needed for activation by STACs, thus one could envision that STACs would have a greater impact on cellular metabolism and less of an impact on p53 stability and the cell cycle. This selectivity may allow the use of STACs in the treatment of SIRT1-dependent metabolic diseases while avoiding some of the adverse pro-oncogenic effects of p53 deacetylation and destabilization.

Of course, full proof of such molecular mechanisms will only come from crystal structure analysis of SIRT1 in the presence and absence of STACs, work that will also allow for the design of more efficacious and diversely targeted STACs. It will be interesting to determine whether SIRT1 STACs or similarly structured compounds could influence other sirtuins. In this regard, glutamate 230 is not conserved in any of the other mammalian sirtuins, suggesting that the allosteric mechanism may work specifically for SIRT1. Overall, these studies provide solid new evidence for a molecular mechanism of action for these compounds, and likely set up the basis for hypothesis-driven pharmacological applications of these STACs in the near future.
